# The burden of diabetes mortality in Finland 1988-2007 - A brief report

**DOI:** 10.1186/1471-2458-11-747

**Published:** 2011-09-30

**Authors:** Kristiina Manderbacka, Riina Peltonen, Seppo Koskinen, Pekka Martikainen

**Affiliations:** 1 Service System Research Unit, National Institute for Health and Welfare, P.O.Box 30, FIN-00271 Helsinki, Finland; 2 Population Research Unit, Department of Social Research, P.O.Box 54, 00014 University of Helsinki, Helsinki, Finland; 3 Department of Health, Functional Capacity and Welfare, National Institute for Health and Welfare, P.O.Box 30, FIN-00271 Helsinki, Finland

## Abstract

**Background:**

Increasing incidence of diabetes has been reported in many countries and the disease burden related to diabetes to be distributed unevenly across the population. Patients with lower socioeconomic position have been reported to have higher diabetes prevalence, higher rates of diabetes related complications and excess mortality. This study examined trends in gender, age and socioeconomic differences in the burden of diabetes mortality in the Finnish population aged 35-80 and potential years of life lost (PYLL) due to diabetes.

**Methods:**

The data consist of an 11% random sample of Finnish residents in 1987-2007 and an 80% oversample of persons who died during those years. We examined diabetes both as underlying and contributory cause. We calculated age-specific and age-standardized diabetes death rates by gender and socioeconomic position using the direct method and PYLL due to diabetes related deaths for 2004-2007.

**Results:**

Diabetes related mortality was higher among older Finns. A clear and systematic socioeconomic pattern was detected among both men and women: the higher the socioeconomic position the lower the mortality. The contribution of diabetes to PYLL was 8% among men and 6% among women. Among women, the contribution of diabetes to PYLL was lower in higher socioeconomic groups, whereas among men, the contribution was similar in all socioeconomic groups.

**Conclusions:**

In order to further reduce the burden of diabetes a better treatment balance to prevent diabetes complications would significantly decrease the burden of diabetes mortality. Use of underlying and contributory causes of death is useful in monitoring trends and sub-group differences in the burden of diabetes.

## Background

Increasing incidence of especially type 2 diabetes has been reported in many countries worldwide [1]. At the same time studies have reported higher rates of premature morbidity and mortality among diabetics than in the general population irrespective of the country, study period or age-group studied [see e.g. [2-7]]. For example, in a meta-analysis including data from 37 studies worldwide the excess risk for fatal coronary heart disease was estimated to be 3.5 among women and 2.06 among men [3]. The disease burden related to diabetes has been reported to be distributed unevenly across the population. Earlier studies have reported higher diabetes prevalence [8], higher rates of diabetes related complications and excess mortality [9-12] among those with lower as compared to those with higher socioeconomic position. For example, a study from ten European countries reported an overall prevalence ratio of 1.6 of diabetes among men with lower secondary education compared to men with tertiary education, among women, the ratio was 2.2. The risk ratio for mortality varied from 1.3 to 5.2 among men and from 2.0 to 10.7 among women in the 1990s and early 2000s [8].

Earlier research on the burden of diabetes mortality has mainly examined mortality among patients with a diagnosis of diabetes and examined diabetes as the underlying cause of death along with other main causes. Whereas different strategies have been used to identify the total diabetes population (e.g., clinical examination, register based data, survey data), part of the diabetes population is likely to be always missed (e.g., undiagnosed cases, persons treated by diet only). Another method used for analyzing the burden of diabetes mortality has been to estimate the number of deaths attributable to diabetes in the general population. Traditionally only the underlying cause of death has been taken into account, but there are some relatively recent studies that also take into account contributory causes of death [13-15]. Few of these studies have examined mortality differences in different population groups, e.g., regionally [14] or by socioeconomic position [13]. However, Miech and colleagues [13] found widening educational differences in diabetes mortality in the U.S. between 1989 and 2005.

The aim of this study is to examine the total burden of diabetes mortality in the general Finnish population by age, gender and socioeconomic position and trends in it from 1988 to 2007. We further aim to analyze socioeconomic differences in potential years of life lost due to diabetes. We assess diabetes both as the underlying and contributory cause of death for the total population aged 35-80 years. In order to evaluate the total burden of diabetes, we analyzed both underlying and contributory causes of death since diabetes associates with several potentially fatal chronic complications, including cardiovascular and renal disease.

## Methods

The data used in the study are based on information from different administrative registers linked with death records by Statistics Finland. These individual level data consist of an 11 percent random sample of the population residing in Finland in 1987-2007 and an 80 percent oversample of persons who died during those years. We used weights in the analyses to take account of this sampling design. Immigrants were dropped out and emigrants censored (1.2% of the sample). In all our analyses diabetes related deaths refer to diabetes as an underlying or a contributory cause, classified by Statistics Finland according to the International Classification of Diseases (ICD). The 9th revision of the classification was used for years 1988-1995 (code 250) and the 10th revision for years 1996-2007 (codes E10-E14).

Information on occupation-based socioeconomic position was available for every 5^th ^year: 1985, 1990, ..., 2005. These were used for the mortality analyses in the years 1988-1990, 1991-1995, ..., 2006-2007 respectively. Four groups were used: (1) upper non-manual, (2) lower non-manual and (3) manual workers and (4) farmers or other entrepreneurs. Those working at home were classified according to the occupation of the head of the household [16]; in the 2000s more than 80% of women with children aged 3+ were employed. The classification we use is also retrospective i.e. information on occupation for the economically inactive population (e.g. pensioners and unemployed) was searched from earlier years. This classification has been used previously for studies of social inequalities in mortality [17]. A small group of students and persons for whom socioeconomic position could not be determined (2% of men, 3% of women) was excluded from the analyses concerning socioeconomic position. Confirmatory analyses based on a three category education variable replicate the main diabetes mortality trends observed for occupation-based socioeconomic position.

For the trend analysis between years 1988-2007 we calculated age-specific diabetes death rates by gender and age-standardized diabetes death rates by gender and socioeconomic position. Standardization was done by using the direct method and using the sum of all person-years in the total population in 5-year age groups as the standard.

The analysis of potential years of life lost (PYLL) between the ages 35 and 80 due to diabetes related deaths by socioeconomic position was performed for the period 2004-2007. PYLL was calculated as the sum of deaths weighted by the difference between age at death and age 80[18]. We calculated 95% confidence intervals for PYLL on the basis of the Taylor linearized variance estimator with Stata version 11.1 [19].

## Results

**Figure 1:**
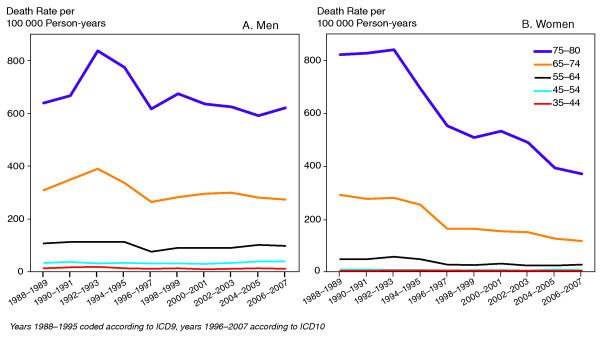
**Diabetes death rates per 100 000 person-years by age in 1988-2007, men (A) and women (B)**.

Altogether 346 878 deaths occurred among men and 235 466 deaths among women during the follow-up period 1988-2007. Numbers for diabetes related deaths were 27 224 and 26 113, corresponding to eight percent of deaths among men and 11 percent among women. Figure 1 shows trends in diabetes related deaths per 100 000 person years by age-group among men and women. A systematic age pattern was detected in diabetes related deaths for both genders (p < 0.001): the older the age-group the higher the mortality. Diabetes related mortality declined during the follow-up period especially among women (p < 0.001), and the decline was specially marked in the oldest age-groups (65+) compared to younger age groups. Diabetes related mortality was relatively uncommon among those younger than 65 years for both genders and showed little decline during the follow-up among men.

**Figure 2:**
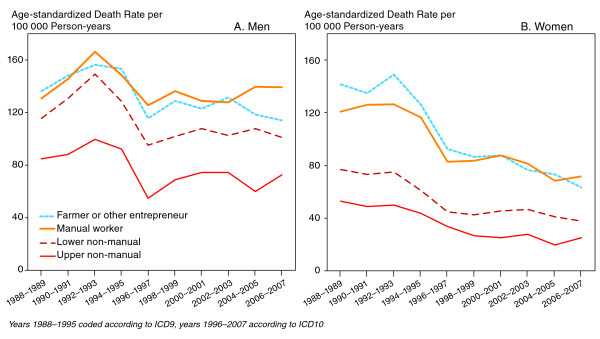
**Age-standardized diabetes death rates per 100 000 person-years by socioeconomic position in 1988-2007, men (A) and women (B) aged 35-80**.

At the beginning of the study period in 1988-1989 those in lower socioeconomic position had higher diabetes mortality than those in higher positions and the differences remained throughout the study period (Figure 2; for men p < 0.05 for the difference between upper non-manual and manual in every period; p < 0.001 for women). Diabetes related mortality among lower white-collar men resembled that of manual men, whereas among women, it was more close to mortality among upper non-manual workers. Mortality among farmers and other entrepreneurs was similar to that among manual workers. Mortality declined in each socioeconomic group over the follow-up period among both genders. Furthermore, absolute differences between socioeconomic groups declined among women but increased among men. However, the relative differences increased among both genders.

Altogether 1439 potential years of life were lost per 100 000 person years due to diabetes related deaths between 2004 and 2007 among men, and 506 years among women. In total, diabetes related mortality constituted eight percent of the total potential years of life lost (PYLL) among men and six percent among women (Table 1). The higher the socioeconomic position the fewer the potential years of life lost. Among men the relative differences between socioeconomic groups in PYLL due to diabetes were similar to those due to other causes. Instead, among women the relative differences were larger in PYLL due to diabetes compared to those due to other causes. Among men, the contribution of diabetes to PYLL from all causes was similar in all socioeconomic groups, but among women, higher socioeconomic position was associated with a lower contribution of diabetes to PYLL.

**Table 1:** Age-standardized potential years of life lost (PYLL) per 100 000 person-years due to diabetes and all causes

		PYLL due to diabetes				PYLL due to all causes				
		
	Person-years, %	PYLL	95% CI	Absolute difference	Relative difference	PYLL	95% CI	Absolute difference	Relative difference	Diabetes PYLL of all PYLL, %
**Men**										
Upper non-manual	18	786	(697-874)	0	1.00	10008	(9661-10355)	0	1.00	7.9
Lower non-manual	19	1230	(1121-1339)	444	1.57	14734	(14307-15161)	4726	1.47	8.3
Manual worker	46	1795	(1710-1880)	1009	2.28	23219	(22858-23580)	13211	2.32	7.7
Farmer or other entrepreneur	17	1350	(1234-1465)	564	1.72	15455	(14988-15923)	5448	1.54	8.7
All	100	1439	(1388-1490)	-	-	17931	(17722-18140)	-	-	8.0
**Women**										
Upper non-manual	16	171	(130-212)	0	1.00	5495	(5229-5761)	0	1.00	3.1
Lower non-manual	43	392	(354-430)	221	2.29	7046	(6864-7228)	1551	1.28	5.6
Manual worker	30	741	(675-807)	570	4.33	10253	(9964-10543)	4759	1.87	7.2
Farmer or other entrepreneur	11	616	(525-707)	445	3.60	8028	(7621-8435)	2533	1.46	7.7
All	100	506	(478-534)	-	-	7855	(7727-7984)	-	-	6.4

## Discussion

This study examined the total burden of diabetes mortality in the Finnish population in 1988-2007. Both underlying and contributory causes were included. A systematic age pattern was detected in diabetes related deaths for both genders: the older the age-group the higher the mortality. Diabetes related mortality declined during the follow-up period especially among women, and the decline was especially large in the oldest age-groups. More research is needed to evaluate the causes of these trends. In line with earlier studies of total mortality among persons with diabetes and studies of diabetes related mortality [9,10,13,20] a clear socioeconomic pattern was detected among the three employee categories (upper non-manual, lower non-manual, manual) among both men and women: the higher the socioeconomic position the lower the mortality. The contribution of diabetes related mortality to potential years of life lost was eight percent among men and six percent among women. Among men, the contribution of diabetes to potential years of life lost was similar in all socioeconomic groups suggesting that diabetes related mortality does not contribute to socioeconomic differences in total mortality. However, among women the contribution of diabetes to potential years of life lost was larger in lower groups.

The longitudinal nature of our individual level data enabled us to analyze trends in diabetes mortality. The Finnish Causes of death register is considered valid and reliable by international standards [21]. Furthermore, the rate of confirmation of the diagnosis by autopsy in Finland is high compared to many other countries (30% for all deaths and about 60% of those of working age) [22]. A limitation of our study is that we use ICD9 for defining the conditions analyzed in 1988-1995 and ICD10 from 1996 onwards. The update in ICD nosology is likely to have at least some contribution to the change in the level of mortality between 1995 and 1996. However, it is not likely to influence gender, socioeconomic or age differences in diabetes related mortality.

It is possible however that our data may still underestimate the number of diabetes deaths. We thus compared the diabetes deaths in our data to those in earlier studies examining mortality among diabetes patients [20,23]. We observed no significant difference between the two approaches in the level of diabetes related mortality, which we consider a validation to both approaches of analyzing diabetes as a cause of death. However, both approaches are still likely to be underestimates to the extent that there are persons suffering diabetes without knowing it and diabetes may be under-recorded in death certificates [24].

## Conclusions

According to our results diabetes related mortality declined especially among older age groups in Finland suggesting improvements in diabetes care in the 1990s and 2000s particularly among women. Since diabetes incidence has increased in Finland and elsewhere [1], improvements in diabetes care have been instrumental in offsetting this change. In order to further reduce the burden of diabetes a better treatment balance of diabetes in order to prevent complications would significantly decrease the burden of diabetes mortality. Use of both underlying and contributory causes of death certification may be useful in monitoring trends and population sub-group differences in the burden of diabetes in order to take into account also the contribution of diabetes to mortality due to its complications.

## Competing interest statement

The authors declare that they have no competing interests.

## Authors' contributions

KM contributed to the conception and design of the study, planning of analyses, and drafted the manuscript. RP contributed to the conception and design of the study, performed the statistical analyses and took part of the revision of the manuscript for important intellectual content. SK contributed to the conception and design of the study, planning of analyses and took part of the revision of the manuscript for important intellectual content. PM contributed to the conception and design of the study, planning of analyses, the collection of the data and took part of the revision of the manuscript for important intellectual content. All authors have read and approved the final manuscript.

## Pre-publication history

The pre-publication history for this paper can be accessed here:

http://www.biomedcentral.com/1471-2458/11/747/prepub
